# Prescription Tablets in the Digital Age: A Cross-Sectional Study Exploring Patient and Physician Attitudes Toward the Use of Tablets for Clinic-Based Personalized Health Care Information Exchange

**DOI:** 10.2196/resprot.3806

**Published:** 2015-10-19

**Authors:** Vishal Patel, Timothy M Hale, Sandeep Palakodeti, Joseph C Kvedar, Kamal Jethwani

**Affiliations:** ^1^ Kaiser Permanente San Francisco, CA United States; ^2^ Connected Health Innovation Partners HealthCare Connected Health Boston, MA United States; ^3^ Massachusetts General Hospital Boston, MA United States; ^4^ Harvard Medical School Boston, MA United States; ^5^ Department of Hospital Internal Medicine Mayo Clinic Rochester, MN United States

**Keywords:** electronic health records, tablets, health education, self-management, primary care, physician patient relationship, digital health, iPad, patient-reported outcome measures

## Abstract

**Background:**

To reduce the cost of health care while increasing efficiency and quality, health systems are seeking innovative means to engage and empower patients. Improved use of information technology and electronic health record (EHR) infrastructure is essential, and required for “meaningful use” as mandated by the federal government. Providing personalized health information using tablets at the point of care could enhance the clinical experience and enable efficient collection of patient reported outcome measures to guide clinical decision making.

**Objective:**

The aim of this study is to explore patient and provider attitudes and interest in a proposed clinic-based tablet system for personal health information exchange. To provide a context to understand patients’ use of tablets during their clinic visit, we also examine patients’ current activities and time spent in the waiting room, and their use of health information resources.

**Methods:**

Surveys were administered to 84 patients in the waiting room of a community health center affiliated with Massachusetts General Hospital (MGH) in Boston, MA. This survey included a vignette and illustration describing a proposed tablet-based system in which the patient, upon sign in at the clinic, receives a tablet loaded with personalized information tailored to their specific medical conditions and preferences. Patients were queried about their interest in such a system in comparison to traditional forms of patient education as well as their current health information seeking behaviors and activities and time spent in the waiting room. Interviews with five MGH-affiliated health care providers were conducted to assess their opinions regarding the proposed tablet system.

**Results:**

The majority (>60%) of patients were “very” or “extremely” interested in the proposed tablet system and thought it would improve their knowledge about their medical condition (60%), assist them in making healthy choices (57%), and help them to feel more comfortable talking with their provider (55%). Patients thought the system would be more motivating, informative, and engaging than traditional printed health education materials. The tablet system was not considered more effective than face-to-face interaction with providers, though 44% thought it would improve their relationship with their physician. Overall, 91% of respondents were willing to learn how to use a tablet and 75% reported being “very” or “extremely” confident they could use one. Four of the five providers believed that the proposed tablet system would improve clinical workflow and patient education. Patients and providers were concerned about privacy and security of data collected using the tablets.

**Conclusions:**

Both patients and providers were highly amenable to integrating tablets into the clinical experience, and tablets may be useful in improving patients’ health knowledge, the collection of patient reported outcome measures, and improved patient-provider communication. Further research into operationalizing such systems and their validation is necessary before integration into standard clinical practice.

## Introduction

Total health care expenditure in the United States is expected to reach US $4.4 trillion dollars by 2018, comprising over a fifth of total gross domestic product (GDP) [1]. Greater than three fourths of this expenditure will be related to chronic medical conditions, which can either be improved or prevented given the appropriate lifestyle modifications [2]. These staggering statistics highlight the importance of promoting behavior change, healthy lifestyles, and chronic disease self-management—not only to help control the rising cost of health care, but also to actively engage patients in their own wellness, reduce morbidity, and improve patient satisfaction and quality of life. Unfortunately, many teachable moments for patients are missed due to increasing educational tasks of health care providers and time constraints [3].

For example, it is well-known that regular physical activity can improve blood pressure, blood glucose control, and quality of life, while lowering harmful cholesterol levels and decreasing cardiovascular events and mortality [4-7]. Although providers understand the importance of their role in engaging patients to participate in regular exercise, they report that the lack of time and competing demands are significant barriers in providing this service and less than one third of primary care visits include any exercise or lifestyle counseling at all [8,9]. In an era when medical trainees have been found to only spend 12% of their time (as little as 8 minutes per patient) examining and talking to patients and more than 40% behind a computer, it is of utmost importance to find innovative solutions to provide patients and providers with a more meaningful interaction [10]. Additionally, with the advancement of the Meaningful Use criteria set forth by the federal government, electronic health records (EHRs) must adapt to provide additional control for patient’s data and demonstrate an improvement in patient outcomes [11].

As described by Sinsky et al [12], we have come to a time in the evolution of EHRs that the power of these technologies to support the needs of primary care providers and patients must be better utilized. New electronic information tools should not only add value to the interaction with a patient-centric design, but must also allow providers the opportunity to improve efficiency and align with the goals of the patient-physician relationship [12]. Primary care is at a turning point in history and recognizing the need to improve these aspects with new technologies will help steer future physicians to this rewarding and much needed profession [12]. One potential method of optimizing workflow, decreasing the burden on providers, and increasing the potency of an office-based visit is by leveraging the use of digital technology, such as tablets, to increase patient knowledge and self-management [3,13-15]. This method also makes more efficient use of the limited time that a provider has with the individual patient in that tablets can be used to collect and integrate pertinent patient data prior to the face-to-face clinical encounter, allowing providers to focus on the interaction with the patient and higher level analysis rather than data entry [16-19].

Since the advent of the extremely popular iPad in 2009, the health care community has widely adopted its use as a reference in clinical practice [20,21]. Touchscreen tablets, however, are also increasingly being employed to provide education to patients when they come into the clinic, rather than just a reference tool for providers [20,22]. Organizations such as the Mayo Clinic and the Cleveland Clinic have developed unique patient education applications on various medical conditions and a quick search of the Apple App Store reveals hundreds of such applications [23,24].

Questions arise as to the ubiquitous use of tablets in patient education and engagement for a variety of reasons. While it has been shown that patients who were older, had lower annual household income, and lower educational attainment had more difficulty using advanced communication technologies, a vast majority (94%) of patients across all socioeconomic status backgrounds rated tablet devices as easy to use [25]. Further, by using audio-visual digital media, even low-literacy patients and those recovering from major surgery could be educated about their medical conditions effectively [26,27].

Although many systems have been devised to apply information technology to engage and educate patients, none have achieved widespread use [13]. In the face of a rapidly evolving health information technology (HIT) infrastructure, it is important to create standardized systems that are easy to use and truly afford patients more access to and control over their health data [28]. Prior studies examining the clinical effects of computer-based education and self-management have shown modest benefits on a range of conditions: blood sugar control in diabetes, weight loss after Roux-en-Y gastric bypass surgery, and adherence to immunosuppressive medications after lung transplantation [29-31]. Utilizing computerized educational tools such as kiosks has also been shown to increase patient knowledge on a range of conditions, including the importance of HIV screening, appropriate antibiotic use for upper respiratory tract infections, and adequate asthma care [32-34]. Although a tablet-based patient education system could have many potential benefits similar to the kiosk model, the form factor lends itself more to rapid adoption and scalability. Currently, there is a paucity of evidence regarding patient and provider perceptions of the benefits and barriers of using tablets in the primary care setting.

With this formative study, we sought to explore both patient and provider interest in using tablets for personalized health information exchange in the primary care setting. We hypothesized that this technology could be a powerful catalyst in transforming the health care experience, creating a platform for just-in-time patient education, intelligent intake exams, and collection of patient-reported outcome measures (PROMs). This strategy also takes advantage of the psychology of physically coming to the clinic where patients give their time and attention in hope of receiving personal health care and advice. We also sought to understand patients’ current activities and the amount of time spent in the waiting room, and their health information use, needs, and preferences. The results of this formative research will help guide the design of future clinic-based HIT systems to increase patient knowledge, engagement, and satisfaction while improving provider efficiency and outreach.

## Methods

### Recruitment

The study was developed at Connected Health Innovation (CHI) in Boston, Massachusetts, and approved by the Institutional Review Board of Partners HealthCare and Massachusetts General Hospital (MGH). A research intern administered the survey portion of the study by approaching patients in the waiting room of primary care physicians at an MGH-affiliated community health center in the greater Boston area. This clinic was selected because it is located in an ethnically and socioeconomically diverse community that is relatively representative of patients in the Boston metropolitan area. Eligibility criteria included age 18-75 years and patient status at the clinic. Due to the formative nature of this study, the survey was available only in English and participation was restricted to patients who could speak English. Participation was voluntary and a small remuneration of US $5.00 cash was provided to patients in appreciation for their time. Of the 194 people approached, 115 (59.3%) agreed to participate and of these, 28 (24%) were excluded for not meeting eligibility criteria. The most common reasons for exclusion were age and inability to speak English (n=24). Of the 87 that met the inclusion criteria, 84 completed and returned the survey.

Our second goal was to gather formative data to highlight key points, both positive and negative, that would be of concern to providers in the creation and implementation of a tablet-based system. Five MGH-affiliated providers were recruited using a snowball sampling method in which each provider interviewed recommended another provider to contact. Five providers were contacted via email and all 5 consented to participate in a semistructured phone interview. Providers did not receive remuneration for their participation.

### Data Collection

**Figure 1:**
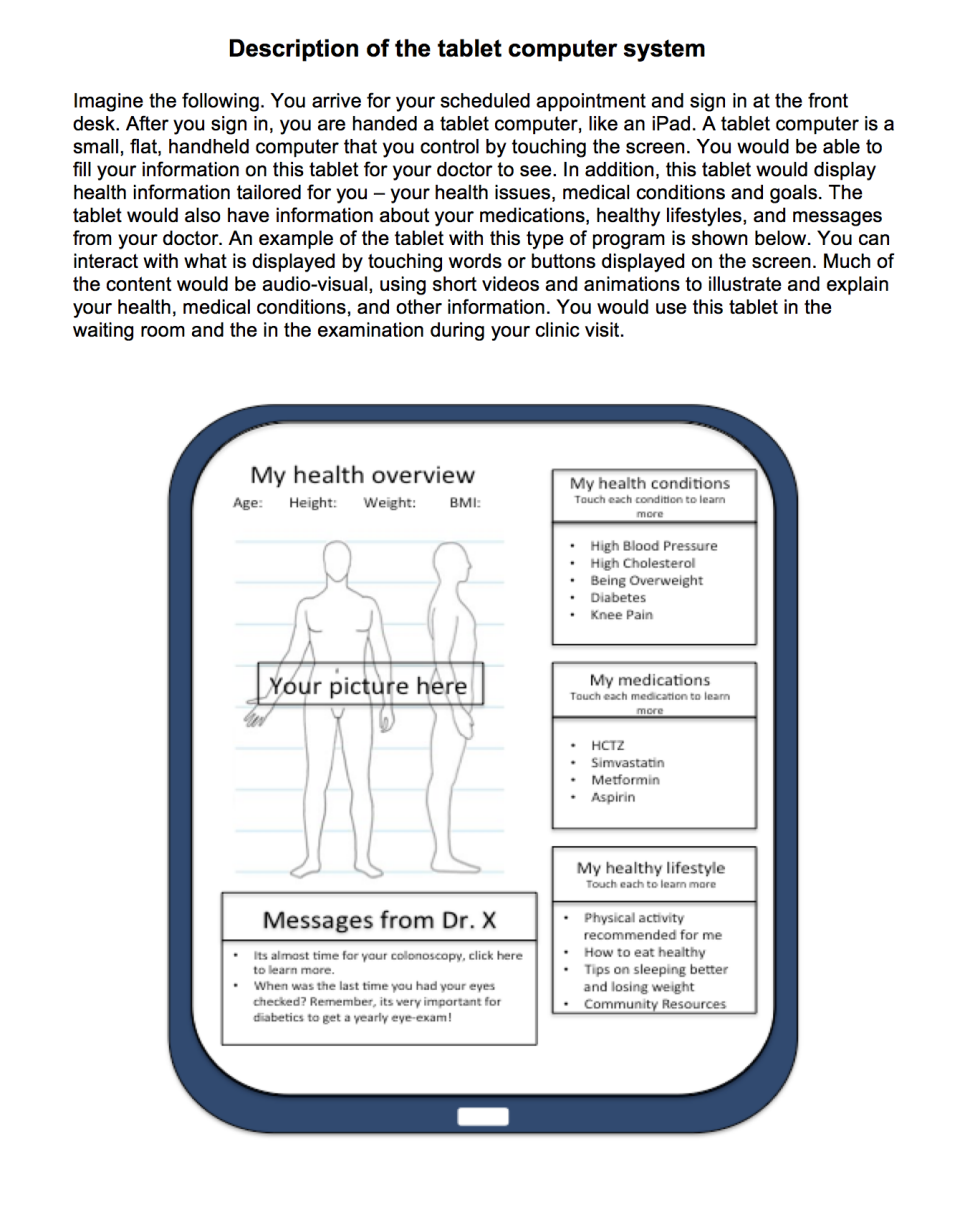
Diagram and description of tablet-based health information exchange system presented to patients.

A 16-page paper survey containing 46 questions was administered to patients and took about 15 minutes for patients to complete (see Multimedia Appendix 1). National surveys were used as the source of questions on technology and Internet use (ie, Pew Research Center) and health information sources, patient-provider communication, health care utilization, and sociodemographics (ie, National Cancer Institute’s Health Information National Trends Survey). The survey included a description and illustration of the user interface of the proposed tablet system (Figure 1) and a series of questions regarding patients’ interest in using this system, types of information they would like to receive, privacy and usability concerns, and attitudes regarding the impact of the system on their health care. Questions about the proposed tablet system and time spent in the waiting room were created by the researchers based on their prior experience studying connected health adoption and use.

A semistructured interview script was created by the research staff to guide the phone interview with providers. The interview covered the practice type and patient characteristics, time and methods used to counsel and educate patients, and perceived efficacy of current education, and patient-provider interactions to improve knowledge and promote healthier behaviors (see Multimedia Appendix 2). Following a description of the proposed tablet system, providers were asked to give their thoughts regarding the proposed system, including general positive or negative reactions, how such a system might be used in the clinic (ie, education, patient-provider communication, decision making, tracking symptoms or patient reported outcomes), potential efficacy, and any concerns on disadvantages or difficulties that might be encountered. Interviews were conducted by a research intern, took about 15 minutes, and an audiorecording was made for transcription and summarizing provider responses.

### Statistical Analysis

Descriptive statistics were generated from the patient surveys using Excel (Microsoft, Redmond, WA, USA) and Stata version 13 (StataCorp, College Station, TX, USA). Transcripts of the interviews with providers were reviewed by the research intern and a research scientist at the Connected Health Innovation (CHI) to summarize providers’ responses to questions and general comments.

## Results

### Patient Characteristics

Characteristics of the patient sample are presented in Table 1. The mean age was 43 years. Thirty-nine percent were male and 72% (60/83) described their race as white, with the largest single minority being Hispanic. A little over half of the sample (57.5%, 46/80) had completed one or more years of college. Most patients rated their health as “good” (37.5%, 30/80) or “very good” (32.5%, 26/80).

**Table 1:** Patient sample characteristics.

Variable		Participants^a^
Age, mean (SD)		43.05 (13.29)
Sex, n (%)		
	Male	31/80 (38.75)
	Female	49/80 (61.25)
Race/ethnicity, n (%)		
	White	60/83 (72.29)
	Hispanic	5/83 (6.02)
	Black	4/83 (4.82)
	Asian	4/83 (4.82)
	Other	10/83 (11.05)
Marital status, n (%)		
	Married or living with partner	45/80 (56.25)
	Divorced, separated, or widowed	11/80 (13.75)
	Single, never married	24/80 (30.00)
Education level, n (%)		
	1st-11th grade	6/80 (7.50)
	12th grade, completed high school, or GED	28/80 (35.00)
	1 to 3 years of college	25/80 (31.25)
	4 or more years of college	21/80 (26.25)
Has a regular health care provider, n (%)		52/81 (64.20)
Number of times saw physician during past year, mean (SD)		3.15 (1.91)
Self-rated health status, n (%)		
	Poor	3/80 (3.75)
	Fair	12/80 (15.00)
	Good	30/80 (37.50)
	Very good	26/80 (32.50)
	Excellent	9/80 (11.25)

^a^Due to missing data (no response to some of the questions on the survey), there is incomplete data on some questions. Thus, only 80 people filled out the question on sex, 83 completed the race question, etc.

Twenty-four percent (17/70) of patients already own a tablet, 79% (61/77) own a laptop or notebook, and 74% (59/80) own a desktop computer. Seventy percent own a smartphone, 14% (11/78) a “feature” phone (a mobile phone that lacks smartphone features), and 65% (50/77) have a wired telephone. An overwhelming majority (91%, 73/80) of patients said they were willing to learn to use a tablet and 76% (63/83) were “very” or “extremely” confident they could use a tablet if they had to. Only 2% (2/83) said they were “not at all confident” they could use a tablet.

### Time Spent in the Waiting Room

The time patients spent waiting to see their doctor during their last visit varied widely with 3 reporting wait times of 90 minutes or longer. The mean wait time was 28.5 minutes with a median of 20 minutes. Taking into account these factors, an estimate of typical wait times is between 20 and 25 minutes.

Fifty-six percent of patients said that the time they spent waiting in the doctor’s office was time *not* well spent. We asked patients to choose from a list of 13 activities all of the things they normally do while waiting to see their doctor. The most common activities were reading the newspaper (63%, 53/84) or sitting quietly (43%, 36/84). A substantial percentage of patients used their personal devices to send or receive text messages (short message service, SMS) (29%, 24/84), browse the Internet (23%, 19/84), or talk on the phone (21%, 18/84). Patients did report spending some time on health-related activities while waiting—24% (20/84) spent some time reading handouts or pamphlets on health topics.

### Health Information Seeking, Sharing Information With Physicians, and Information Needs

Eighty-nine percent (74/83) of patients said they had at one time looked for information about health or medical topics. When these patients last looked for health information, the overwhelming majority turned first to the Internet (83%, 52/63), with doctors and other health care providers being placed a distant second (8%, 5/63). During their most recent search, patients were most often seeking information for themselves (56%, 43/77) or for both themselves and someone else (29%, 22/77). Only 13% (10/77) of patients were seeking health information for someone else. Based on patients’ experiences during their most recent search for health information, 53% (40/75) were “concerned about the quality of the information” they found. A substantial proportion of patients reported that finding the health information they needed took “a lot of effort” (30%, 23/77) and was “hard to understand” (24%, 18/75).

Although doctors and other health care providers placed a distant second to the Internet as a source of health information during patients’ last search, the gap narrowed when patients were asked where they would turn first if they had a strong need to get health or medical information. Fifty-seven percent (44/77) said they would use the Internet and 29% (22/77) said they would turn first to their doctor or health care provider. About 39% (31/80) of patients said they talked with their doctor or health care provider in the last 12 months about the information they found online. However, provider interest in hearing about this information was mixed. Patients said that 40% (17/43) of providers were “not interested at all” or “a little interested”, and 60% (26/43) were “somewhat” or “very” interested.

Patients gave doctors and other health care providers high marks for providing clear explanations on health care issues, but lower marks for dealing with patient’s emotions and feelings, time spent with patients, and providing enough opportunities to ask questions. Patients said that 92% (76/83) of providers “usually” or “always” explained things in a way patients said they could understand, and 88% (73/83) “usually” or “always” made sure the patient understood what they needed to know to take care of their health. However, patients reported that 23% (19/82) of providers “never” or only “sometimes” gave enough attention to their feelings and emotions, helped them to deal with feelings of uncertainty, or spent enough time with them during the clinic visit. Twenty-three percent (19/83) of patients said that providers “never” or only “sometimes” gave them enough time to ask all of the health-related questions they had.

Patients were asked about their preferred setting to learn about their health and what types of information they were most interested in receiving. Fifty-three percent (36/69) stated they would prefer to receive this information during their visit to the clinic, either before (28%, 19/69) or after (25%, 17/69) seeing their provider. Forty-seven percent (33/69) of patients preferred reviewing clinical information at home either on their own time (17%, 12/69), before (14%, 10/69), or after (16%, 11/69) their appointment. When asked to select one or more of 6 types of health information, patients were most interested in receiving information on specific health issues (78%, 64/82), test results (68%, 56/82), medications and side effects (59%, 48/82), general health and wellness (55%, 45/82), chronic pain management (38%, 31/82), and community health resources (33%, 27/82).

### Patient Interest and Attitudes Toward the Proposed Tablet-Based System

Patients expressed a high level of interest in the proposed tablet-based system, with 64% (54/84) saying they were “extremely” or “very” interested and only 5% (4/84) saying they were “not interested at all”.

Compared to other methods to disseminate health information, patients believed the tablet system would be more motivating, engaging, and informative than printed materials, websites, and emails. For example, compared to printed materials, the tablet system was considered more motivating (80%, 66/83), engaging (86%, 69/80), and informative (78%, 63/81). However, the tablet system was not considered to be superior to face-to-face information exchange with providers –patients were about equally divided in choosing between the tablet or providers as more motivating (53%, 44/83 choosing tablets), more informative (51%, 40/79 choosing providers), and more engaging (54%, 43/80 choosing providers).

Patients believed the proposed tablet system would have positive effects on their health care. The majority of patients agreed that using the tablet system during clinic visits would improve their knowledge of their medical condition (60%, 49/81), would assist them in making healthy lifestyle choices (57%, 47/82), and help them to feel more comfortable talking with their provider about their medical condition (55%, 45/82). Patients were less sure of whether the tablet system would improve their relationship with their provider. Forty-four percent (36/82) agreed that the tablets improve patient-provider relationships, but 33% (27/82) neither agreed nor disagreed with this statement.

The primary concern patients expressed were regarding privacy issues. Thirty-three percent (27/81) said they were concerned about privacy using the proposed tablet system. Typical responses to an open-ended question about privacy issues were concerns of “who will have access to my information” and people “hacking into the system.”

### Provider Interviews

The characteristics of providers and their description of the types of patients they treat are presented in Table 2. Providers interviewed were a diverse group, consisting of 2 physicians (ie, internal medicine, general practitioner), a psychiatrist, a nurse practitioner working in a primary care clinic, and a registered nurse who directs a wellness center. Three providers were located at the same community health center where patients were recruited. Four of the 5 providers treat a diverse patient population with the internal medicine physician noting that the patients they see are mostly older, well-educated, women.

**Table 2:** Provider sample characteristics and the types of patients they see.

		Provider 1	Provider 2	Provider 3	Provider 4	Provider 5
Practice		Internal medicine	General practitioner	Psychiatrist	Nurse practitioner, primary care	Registered nurse, wellness center director
Clinical setting		Wellness center	Community health center	Wellness center	Community health center	Wellness center located at a community health center
Patients						
	Medical conditions	Stress-related medical conditions	Diverse, chronic conditions	Range of psychiatric conditions, many with severe depression	Diverse, chronic conditions	Chronic conditions and chronic pain
	Age, years	40-60	Adults	Adults	Adults	Adults
	Sex	Female	Female	Female	Diverse	Female
	Race/ethnicity	Diverse	Diverse	Diverse	Diverse	Diverse
	Education	Higher levels	All levels	Clinic practice, all levels; private practice higher levels	All levels	Lower levels
	Income	All levels	All levels	Clinic practice, all levels; private practice higher levels	All levels	Lower levels

A majority of providers interviewed (4 of 5) reacted enthusiastically to the idea of incorporating tablets for health information exchange into their clinical workflow. Physicians reported that they spent nearly 25-50% of their time counseling and educating patients on individual lifestyle and behavioral modifications. A majority of providers (4 of 5) are using traditional printed materials as a means to disseminate health communication materials to patients and thought the tablet system would enhance multiple aspects of the clinical encounter. Providers thought that using the tablet system would allow for more personalized content delivery with the possibility of using easy-to-understand, modular audio-visual material. Furthermore, they thought that this content would prompt patients to think about their health issues prior to their clinical encounter and allow patients more time to assimilate their health information with additional context provided by the physician as needed.

Given that data collected with the tablets could be used in the patients’ longitudinal EHR, providers were also interested in using the tablets to gather patient information as a part of the clinical history or intake exam. For example, Provider 3 suggested that the tablet system could incorporate validated visual analog scales to monitor depressive symptoms, which could then be use to track the efficacy of therapy. This would contribute clinically valuable patient-generated health data to the patient’s record as a measure of outcomes. Additionally, providers indicated that the flexibility of such a platform could be used to provide tailored health information content through the use of disease or medical condition specific modules.

The primary barriers with using tablets in the clinic as perceived by providers centered on 3 areas: usability by various patient populations, assurance of patient privacy, and the physical maintenance of tablets in the clinic. One provider was concerned that using a technology-based educational medium would be difficult for certain patients, particularly those who were elderly, had lower socioeconomic status and literacy, and those who were recent immigrants. Additional concerns were for users of the tablet system with movement disorders, chronic pain, and difficulties with vision/hearing, which would prevent them from effectively using the tablet interface. Providers also expressed a need to ensure patient privacy. For example, Provider 5 suggested the use of privacy screens and personal headphones for audio/visual content. Finally, physically keeping the tablets in the clinic as well as ensuring their distribution and return along with ancillary devices (headphones, chargers, privacy screens, etc) was a potential difficulty in implementation, creating additional tasks for support staff.

## Discussion

### Principal Findings

The goal of this study was to explore patients’ and providers’ attitudes regarding a proposed tablet-based personalized health care information exchange system in the waiting room and during the clinical encounter. We found that patients and providers were receptive to the idea of using tablets. A majority of patients believed the tablet system would have positive effects on their health knowledge, assist in making decisions regarding their health, and help them to feel more comfortable communicating with their provider. Patients thought the proposed system would be more engaging, motivating, and informative than other communication channels. Providers thought the tablet system would enable more personalized delivery of health education content to patients and the collection of patient-reported data for use during the clinical encounter. Barriers expressed by both patients and providers were concerns regarding the privacy and security of information collected using a tablet system.

Engaging patients in their health and medical care is understood to be key to achieving better health outcomes and higher patient satisfaction with care [35-37]. However, efforts to improve patient engagement in clinical settings are labor intensive and difficult to implement [38]. A point of care tool that delivers information, prompts patients to take action or change behaviors, and supports patient-provider communication and shared decision making may be the most effective model to improving engagement [38]. The time patients spend in the waiting room is an opportunity for them to learn about their health and make decisions regarding their medical treatment [39]. We found that patients spend about 20-25 minutes in the waiting room and that only 1 in 4 makes use of this time to learn about their health. Additionally, about half of patients preferred to receive health information just before or after their appointment, rather than outside of the clinic. This makes the waiting room an excellent opportunity to provide patients with personalized health information and implement patient-centric interventions into the clinical workflow.

Patients responded positively to the proposed tablet system and thought it would help them to improve their knowledge, assist in making decisions about their health, and feel more comfortable communicating with their providers. A similar tablet system to the one we proposed was found to have a positive effect on patients’ likelihood of asking questions of their providers. Hess et al [40] found that patients randomized to the group using the tablet to receive personalized health information and feedback were more likely to ask questions about mental health issues and a larger, but not statistically significant, percentage initiated some type of discussion with their provider, compared to controls. Unfortunately, a limitation of this pilot study is the cross-sectional design in which patients were asked about their thoughts regarding the proposed tablet system, rather than interventional study design in which subjects’ use of a tablet system is assessed over time. Additional research is thus needed to examine how repeated use and reinforcement of educational information and decision aids delivered by a tablet system might affect patient knowledge and patient-provider interactions.

Efforts to improve patient engagement most often employ educational materials to increase patients’ knowledge and to support shared decision making between patients and providers [41]. Although these materials have been found to be efficacious in improving patient engagement and shared decision making [42], few patients receive these materials during a primary care visit. A 21-month study of 5 primary care practices in California found that only 10% of eligible patients received targeted educational materials about screening tests [41]. The primary barrier cited by physicians that limit the use of educational materials and patient-provider discussions on treatment decisions is time constraint [41]. Interestingly, Lin et al found that physicians who made greater use of educational materials as decision aids reported that it saved them time because patients could review the information before the clinical encounter. The providers we interviewed thought the tablet system would be helpful in delivering personalized information and prompt patients to think about their health and medical treatment outside of the clinical encounter. Additionally, providers were interested in the capability to collect patient-reported outcomes while in the waiting room. A tablet system could automate the data entry, scoring, and analysis of this data, which could then be used to guide the clinical encounter and populate the EHR. This capability may improve workflow efficiency and increase the amount of face-to-face time between provider and patient [16]. Tablets used in an inpatient setting have been found to reduce the time required to check the EHR and increase the time providers spend with patients [18].

Providers expressed some concerns about the feasibility of using tablets in the clinic. One concern was coordinating the distribution and return of equipment. Key to the successful integration of a tablet-based system in the clinical workflow is the adoption by the nonphysician clinic staff who will most likely be responsible for distributing, collecting, and maintaining the tablets. Previous research examining the distribution of decision support aids designed to improve patient engagement, increase knowledge, and support shared decision making-the same goals as those of the proposed tablet system-found that clinic staff were willing to take on this task and were most effective in distributing materials to patients [41]. Successful implementation will also require educating staff and providers on the benefits of a tablet system and how it benefits patients, providers, and contributes to achieving long-term institutional goals. Pilot studies that examine integrating the tablet system into the clinical workflow will be needed to understand how to mitigate barriers if the program is to be scaled successfully.

A second concern of providers was that some patients may not be able to make effective use of tablet-based systems. Research has found that patients who are older and with lower levels of income and education have more difficulty using tablets [25]. However, we found that patients were optimistic about their ability to use the proposed tablet system. Most patients were very or extremely confident they could use a tablet and greater than 90% (73/80) were willing to learn how to use one if they had to. Only 5% (4/84) of patients expressed no interest at all in using the proposed system. Other evidence suggests that concerns about older adults’ use of technology are diminishing. A study investigating the use of tablets among older adults recovering from cardiac surgery found no evidence that older adults were “technophobic”—unwilling or unable to use the tablets [43]. A recent Pew report finds that older adults are rapidly adopting some types of technology, and although this group lags other segments of the population on some types of technology use, they are more likely to own a tablet than a smartphone [44]. This report also echoed the concerns raised by the providers we interviewed; that aging and health-related limitations in sensory and cognitive functioning can make using technology difficult. Continued research and development efforts are needed to ensure that new HIT systems are accessible and usable by patients despite physical and cognitive limitations.

A concern raised by both patients and providers was the issue of privacy and security. These concerns may negatively impact patient and provider trust and adoption, and poses a risk to the success of a tablet system, and more broadly, to the expanded use of HIT [45]. Privacy and security concerns are often noted as a barrier to HIT adoption [46,47]. However, patients may be less concerned about privacy than providers and are generally willing to use technology when they view the benefits as outweighing the risks [48]. For example, in a study using focus groups to explore patients’ attitudes on technology’s future role in health care, researchers found that patients were less concerned about privacy and more concerned with making use of technology to improve their access to relevant medical information, communicate with their provider, and making data available to providers in an emergency [49]. Similarly, despite 33% (27/81) of patients expressing concerns about privacy, we found that 64% (54/84) were extremely or very interested in using the proposed tablet system. Ensuring high levels of trust and the success of a tablet system will require additional research to understand patients’ and providers’ concerns and building a platform that meets technical standards and regulations.

### Limitations

Although the methods used in this study were adequate for collecting data to explore the potential for creating a tablet-based health information system for use in primary care settings, it is not without limitations. First, patients were recruited from only 1 site, a community health center affiliated with a large teaching hospital in the northeast. Although this site was selected due to the ethnic and socioeconomic diversity of the community and patients, this setting is not necessarily representative of patients, physicians, or clinics in the United States. A second limitation of this study is the small sample size which limits the coverage of all types of patients and thus, the generalizability to all patients who receive care at the clinic. Third, findings on provider attitudes are based on a very small sample of 5 providers recruited using a nonprobability, snowball sampling methodology, and should not be interpreted as representative of physicians across specialties, clinics, or regions in the United States. Finally, patients and providers responded based on an illustration and explanation of a proposed tablet system and provided initial reactions to this concept. Use of a functioning system, with its own unique features and user experience, would elicit much different responses.

Ultimately, the goal of this research is to develop and implement new HIT to improve patient engagement and self-management of patient medical conditions, while also improving the efficiency and effectiveness of health care delivery. Future research should support an “agile development” process to guide building a tablet system. In support of this goal, future research is needed to move beyond the results of this exploratory study to understand the specific features and functionality patients and providers require in a tablet system. This could take several stages. First, additional formative research is needed to collect data on these features from a more diverse sample of patients and physicians. Second, after identifying key features, these should be confirmed across potential users using questionnaires and sampling methods to collect data that are representative of the patients, providers, and the clinics and communities they serve. Third, wire-frame mock-ups of the tablet application, or early stage “alpha” versions should be developed and tested with small groups of patients and providers. Through an iterative process of development, testing, and redesign a “beta” version of the tablet application can be tested in a small feasibility study and if successful, in a larger study to evaluate effectiveness.

### Conclusion

Patients and providers were highly amenable to integrating tablets into the clinical experience, and it may be useful in improving patient-provider communication, patients’ health knowledge, and the collection of PROMs. Further research into operationalizing such systems and their validation with patient outcomes is necessary before integration into standard clinical practice.

## Appendix

Multimedia Appendix 1 Survey Instrument.

Multimedia Appendix 2 Provider Interview Guide.

## Abbreviations

AV: audio-visual
CHI: Connected Health Innovation
EHR: electronic health record
GDP: gross domestic product
HIT: health information technology
MGH: Massachusetts General Hospital
PROM: patient-reported outcome measure
