# Supplementary material for: Prescription Tablets in the Digital Age: A Cross-Sectional Study Exploring Patient and Physician Attitudes Toward the Use of Tablets for Clinic-Based Personalized Health Care Information Exchange
Source: JMIR Res Protoc. 2015 Oct 19;4(4):e116. doi: 10.2196/resprot.3806 (PMC4704891; doi:10.2196/resprot.3806)
Supplement: Multimedia Appendix 1 [file resprot_v4i4e116_app1.pdf]

Technology and Healthcare Survey

Participant ID:________________________
(to be completed by research staff)

Date:________________________________
(to be completed by research staff)

Version 09-16-2012

Section A: Technology Use

1. Do you own any of the following devices?
(*For each item, please circle the number for your answer.*)

	Yes	No
**a. A desktop computer.**	1	2
**b. A laptop computer or netbook.**	1	2
**c. A tablet computer like an iPad.**	1	2
**d. A cell phone that can only make calls or send text messages (e.g. flip phone, standard cell phone).**	1	2
**e. A smartphone like an iPhone or Android phone (e.g., a cell phone that can be used to access the Internet and run apps).**	1	2
**f. A landline telephone.**	1	2

2. Do you ever go online to access the Internet or World Wide Web, or to send and receive email? *(Circle the number of your answer.)*

1 Yes

2 No [skip to question 4]

3. When you use the Internet, do you access it through…
(*For each item, please circle the number for your answer.*)

	Yes	No
**a. A regular dial-up telephone line.**	1	2
**b. Broadband such as DSL, cable or FiOS.**	1	2
**c. A cellular network (e.g., phone, 3G/4G)**	1	2
**d. A wireless network (Wi-Fi)**	1	2

4. **How familiar are you with the following Internet-related items? Please choose a number between 1 and 5 where 1 represents having "no understanding" and 5 represents having a "full understanding" of the item. (*For each item, please circle the number for your answer.*)**

	None	A little	Some	Good	Full
**a. Advanced search**	1	2	3	4	5
**b. PDF**	1	2	3	4	5
**c. Spyware**	1	2	3	4	5
**e. Wiki**	1	2	3	4	5
**e. Phishing**	1	2	3	4	5

Section B: Health Information Seeking

5. **Have you ever looked for information about health or medical topics? *(Circle the number of your answer.)***

1 Yes

2 No [skip to question 9]

6. **<u>The most recent time</u> you looked for information about health or medical topics, where did you go first? *(Circle the number of only ONE answer.)***

1 Books

2 Brochures, pamphlets, etc.

3 Non-profit organization

2 Family

3 Friend/Co-worker

4 Doctors or health care provider

5 Internet

6 Partners Patient Gateway

7 Library

8 Magazine or newspaper

9 Telephone information number

10 Other, please specify: ______________________________

7. **<u>The most recent time</u> you looked for information about health or medical topics, what was it for? *(Circle the number of your answer.)***

1 Myself

2 Someone else

3 Both myself and someone else

4 Neither/ General curiosity

8. Based on the results of <u>your most recent search</u> for information about health or medical topics, how much do you agree or disagree with <u>each</u> of the following statements? (*For each item, please circle the number for your answer.*)

	Strongly agree	Somewhat agree	Somewhat disagree	Strongly disagree
**a. It took a lot of effort to get the information you needed.**	1	2	3	4
**b. You felt frustrated during your search for the information.**	1	2	3	4
**c. You were concerned about the quality of the information.**	1	2	3	4
**d. The information you found was hard to understand.**	1	2	3	4

9. Overall, how confident are you that you could get advice or information about health or medical topics if you needed it? *(Circle the number of your answer.)*

1 Not confident at all

2 A little confident

3 Somewhat confident

4 Very confident

5 Completely confident

10. Imagine that you had a strong need to get information about health or medical topics. Where would you go first? *(Circle the number of only ONE answer.)*

1 Books

2 Brochures, pamphlets, etc.

3 Non-profit organization

2 Family

3 Friend/Co-worker

4 Doctors or health care provider

5 Internet

6 Partners Patient Gateway

7 Library

8 Magazine or newspaper

9 Telephone information number

10 Other, please specify: ______________________________

Section C: Using Technology to Access Health Information & Services

11. <u>In the past 12 months,</u> have you used the Internet to look for health or medical information for yourself? ***(Circle the number of your answer.)***

1 Yes

2 No

12. In the last 12 months, have you used the Internet for any of the following reasons?
(*For each item, please circle the number for your answer.*)

	None	About once a year	A few times a year	About once month	About once a week	About once a day
**a. Participated in an online support group for people with similar health or medical issue.**	1	2	3	4	5	6
**b. Used email or the Internet to communicate with a doctor or doctor's office.**	1	2	3	4	5	6
**c. Used a website to help with your diet, weight, or physical activity.**	1	2	3	4	5	6
**d. Looked for a healthcare provider.**	1	2	3	4	5	6
**e. Downloaded health-related information to a mobile device, such as an MP3 player, cell phone, tablet computer or electronic book device.**	1	2	3	4	5	6
**f. Visited a social networking site, such as Facebook or LinkedIn to read and share about medical topics.**	1	2	3	4	5	6
**g. Kept track of personal health information such as care received, test results, or upcoming medical appointments.**	1	2	3	4	5	6
**h. Looked for health or medical information for someone else.**	1	2	3	4	5	6

13. In the past 12 months, have you talked to a doctor, nurse or other health professional about any kind of information you have gotten from the Internet?
(Circle the number of your answer.)

1 Yes

2 No [skip to question 15]

14. In the past 12 months, when you talked with a health care profession, how interested were they in hearing about the information you found on the Internet?
(Circle the number of your answer.)

1 Not at all interested

2 A little interested

3 Somewhat interested

4 Very interested

15. Have you ever heard of Patient Gateway?
(Circle the number of your answer.)

1 Yes

2 No [skip to question 18]

16. <u>In the past 12 months,</u> have you used Patient Gateway?
(Circle the number of your answer.)

1 Yes

2 No [skip to question 18]

17. <u>In the last 12 months,</u> have you used the Patient Gateway for any of the following reasons?
(*For each item, please circle the number for your answer.*)

	None	About once a year	A few times a year	About once month	About once a week	About once a day
**a. Request an appointment.**	1	2	3	4	5	6
**b. Request a referral to see a specialist.**	1	2	3	4	5	6
**c. Communicate with your doctor or doctor's office.**	1	2	3	4	5	6
**d. View test results or radiology reports.**	1	2	3	4	5	6
**e. View your electronic medical record.**	1	2	3	4	5	6
**f. Check for upcoming, scheduled appointments**	1	2	3	4	5	6
**g. Renew a prescription.**	1	2	3	4	5	6
**h. Review your medication and allergies record.**	1	2	3	4	5	6
**i. Search for medical information using the Health Library.**	1	2	3	4	5	6

Section D: Clinic Based Technology

18. How much do you agree or disagree with <u>each</u> of the following statements?
(*For each item, please circle the number for your answer.*)

	Strongly agree	Somewhat agree	Somewhat disagree	Strongly disagree
**a. I am willing to try new technology.**	1	2	3	4
**b. I generally feel confident using new technology.**	1	2	3	4
**c. I worry about security issues of sending health information by the Internet.**	1	2	3	4
**d. It is easy for me to follow instructions and set up new technology.**	1	2	3	4
**e. I have no difficulty setting up computers or Internet modems.**	1	2	3	4

Now we would like to ask you a few questions about how you might make use of a new technology designed to allow you to view health information customized for you and your needs during a visit to see a doctor. Below, we describe how this technology will work. Please read this description and answer the questions that follow.

Description of the tablet computer system

Imagine the following. You arrive for your scheduled appointment and sign in at the front desk. After you sign in, you are handed a tablet computer, like an iPad. A tablet computer is a small, flat, handheld computer that you control by touching the screen. You would be able to fill your information on this tablet for your doctor to see. In addition, this tablet would display health information tailored for you – your health issues, medical conditions and goals. The tablet would also have information about your medications, healthy lifestyles, and messages from your doctor. An example of the tablet with this type of program is shown below. You can interact with what is displayed by touching words or buttons displayed on the screen. Much of the content would be audio-visual, using short videos and animations to illustrate and explain your health, medical conditions, and other information. You would use this tablet in the waiting room and the in the examination during your clinic visit.

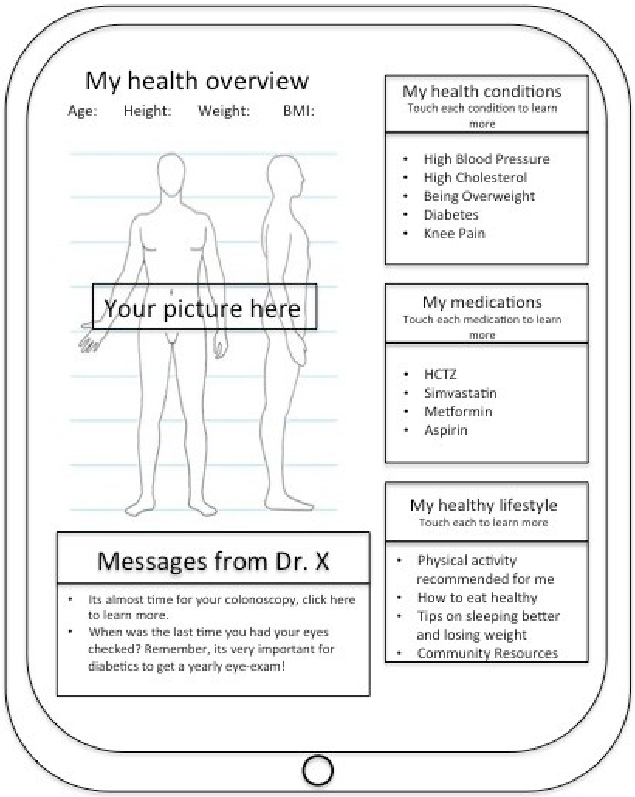

19. **Based on this description, how interested do you think you would be in using this program?** ***(Circle the number of your answer.)***

1 Extremely interested

2 Very interested

3 Somewhat interested

4 A little interested

5 Not interested at all

20. **Would this type of interaction be more *motivating* than the following types of health information distribution? (*For each item, please circle the number for your answer.*)**

	Yes	No
**a. Pamphlets and printed material given to me by my physician**	**1**	**2**
**b, Information that is provided on websites that I would view at home**	**1**	**2**
**c. Face to face interaction with a healthcare provider**	**1**	**2**
**d. Information that is sent to me via e-mail**	**1**	**2**
**e. Information on notice boards and posters in the clinic**	**1**	**2**

21. Would this type of interaction be more *informative and memorable* than the following types of health information distribution? (*For each item, please circle the number for your answer.*)

	Yes	No
**a. Pamphlets and printed material given to me by my physician**	**1**	**2**
**b. Information that is provided on websites that I would view at home**	**1**	**2**
**c. Face to face interaction with a healthcare provider**	**1**	**2**
**d. Information that is sent to me via e-mail**	**1**	**2**

22. Would this type of interaction be more *engaging* than the following types of health information distribution? (*For each item, please circle the number for your answer.*)

	Yes	No
**a. Pamphlets and printed material given to me by my physician**	**1**	**2**
**b. Information that is provided on websites that I would view at home**	**1**	**2**
**c. Face to face interaction with a healthcare provider**	**1**	**2**
**d. Information that is sent to me via e-mail**	**1**	**2**

23. What type of information would you like to receive through a system like this?
(Circle the number for all that apply.)

1 General information relating to healthy lifestyles, behaviors, and exercise
2 Information about how to manage my medical conditions
3 Information regarding the medications I take and their effects/side effects
4 Information regarding my personal health trends
5 Information linking me to community groups and resources to help me stay healthy
6 Personal messages from my physician

24. How much do you agree or disagree with the following statements?
(*For each item, please circle the number for your answer.*)

	Strongly agree	Somewhat agree	Neither agree nor disagree	Somewhat disagree	Strongly disgree
**a. Using a tablet computer during my visits to see the doctor will improve my relationship with my doctor.**	1	2	3	4	5
**b. Using the tablet computer during my visits to see the doctor will help me to feel more comfortable in talking with my doctor about my medical condition.**	1	2	3	4	5
**c. Using a tablet computer during my visits to see the doctor will improve my knowledge of my medical conditions.**	1	2	3	4	5
**d. Using a tablet computer in the proposed method will assist me in making healthy lifestyle choices.**	1	2	3	4	5

25. Do you have any concerns about privacy issues using a system like this?
(Circle the number of your answer.)

1 Yes [if yes, explain in the box below]

2 No

26. Do you have any concerns about actually operating the tablet for this application?
(Circle the number of your answer.)

1 Yes [if yes, explain in the box below]

2 No

27. Would you be willing to learn how to use a touchscreen tablet?
(Circle the number of your answer.)

1 Yes

2 No

28. How confident are you that you could use a tablet computer if you had to?
(Circle the number of your answer.)

1 Extremely confident

2 Very confident

3 Somewhat confident

4 A little confident

5 Not at all confident

29. The last time you visited the doctor, about how many minutes did you wait in the waiting room before you saw the doctor?

__________ ***(Write in number of minutes.)***

30. What do you normally do while waiting in the waiting room to see your doctor?
(circle the number for all that apply)

1 Read the newspaper, magazines, or books
2 Chat on the phone
3 Talk to other patients
4 Send or receive messages
5 Listen to music on your phone or other device
6 Watch videos on your phone or other device
7 Play games on your phone or other device
8 Surf the internet
9 Use an e-Reader (Kindle, etc.)
10 Check your email
11 Watch TV on the one provided in the waiting room
12 Read pamphlets, handouts, or other information about health available in the waiting room or provided to you by your doctor.
13 Sit quietly

31. Do you think your time is well spent while waiting in the doctor's office?
(Circle the number of your answer.)

1 Yes
2 No

32. In what setting do you prefer to learn about your health?
(Circle ONE the number of ONE answer.)

1 While waiting in the clinic to be seen by your physician
2 After being seen by the physician and while still in the clinic
3 At home before the doctors visits
4 At home after the doctor's visit
5 At home on your on time

33. What types of health information are you most interested in receiving?
(Circle the number for all that apply.)

1 General information about health and wellness
2 Information about my specific health issues

3 Information about community resources that will help be stay healthy

4 Information about the medications I take and their side effects

5 Information about medical test results

6 Information about how to manage chronic pain

Section E: Your Health Care and Health Status

34. Not including psychiatrists and other mental health professionals, is there a particular doctor, nurse, or other health professional that you see most often?
(Circle the number of your answer.)

1 Yes

2 No

35. <u>In the past 12 months</u>, not counting times you went to an emergency room, how many times did you go to a doctor, nurse, or other health professional to get care for yourself?
(Circle the number of your answer.)

1 None

2 1 time

3 2 times

4 3 times

5 4 times

6 5-9 times

7 10 or more times

36. The following questions are about your communication with all doctors, nurses, or other health professionals you saw <u>during the past 12 months.</u>
(*For each item, please circle the number for your answer.*)

How often do they do each of the following:	Never	Sometimes	Usually	Always
**a. Give you the chance to ask all the health-related questions you had?**	1	2	3	4
**b. Give the attention you needed to your feelings and emotions?**	1	2	3	4
**c. Involve you in decisions about your health care as much as you wanted?**	1	2	3	4
**d. Make sure you understood the things you needed to take care of your health?**	1	2	3	4
**e. Explain things in a way you could understand?**	1	2	3	4

**f. Spend enough time with you?**	1	2	3	4
**g. Help you deal with feelings of uncertainty about your health or health care?**	1	2	3	4

37. How true is the following statement? "Ultimately, my doctor is the person who is responsible for managing my health conditions."
(Circle the number of your answer.)

1 Not at all true
2 A little true
3 Somewhat true
4 Very true

38. How true is the following statement? "Ultimately, I am the person who is responsible for managing my health conditions."
(Circle the number of your answer.)

1 Not at all true
2 A little true
3 Somewhat true
4 Very true

39. Overall, how confident are you about your ability to take good care of your health?
(Circle the number of your answer.)

1 Not confident at all
2 A little confident
3 Somewhat confident
4 Very confident
5 Completely confident

40. In general, would you say your health is...
(Circle the number of your answer.)

1 Excellent
2 Very Good
3 Good
4 Fair
5 Poor

Section F: Demographics

41. What is your age? __________ ***(Write in number of years.)***

42. What is your gender?
(Circle the number of your answer.)

1 Male
2 Female

43. What is your marital status?
(Circle the number of your answer.)

1 Married
2 Living with partner
3 Divorced or separated
4 Widowed
5 Single, never been married

44. What is the highest grade at school or years in college that you have completed?
(Circle the number of your answer.)

1 1st – 8th grade
2 9th – 11th grade
3 12th grade, completed high school, or GED
4 1 to 3 years of college
5 4 or more years of college

45. Are you Hispanic or Latino?
(Circle the number of your answer.)

1 Yes
2 No

46. Which one or more of the following would you say is your race?
(Circle the number of your answers, more than one may apply.)

1 American Indian or Alaska Native
2 Asian
3 Black or African American
4 Native Hawaiian or other Pacific Islander
5 White
6 Other, please specify: ______________________________

Thank you for taking the time to complete this survey!
