# Supplementary material for: Prescription Tablets in the Digital Age: A Cross-Sectional Study Exploring Patient and Physician Attitudes Toward the Use of Tablets for Clinic-Based Personalized Health Care Information Exchange
Source: JMIR Res Protoc. 2015 Oct 19;4(4):e116. doi: 10.2196/resprot.3806 (PMC4704891; doi:10.2196/resprot.3806)
Supplement: Multimedia Appendix 2 [file resprot_v4i4e116_app2.pdf]

Health Care Provider Interview Guide
Version: 08/31/2012

1. **Introduction**
   a. Introduction by researcher/interviewer.
   b. Review study purpose and goals
   c. Review risk and benefits to participation in the study, questions that will be asked, recording and transcription of the interview, voluntary participation, and no renumeration for providers.
   d. Answer any remaining questions about the study.
   e. Obtain verbal consent prior to beginning the interview.
2. **Background**
   a. General description of health care provider's background and practice.
   b. Characteristics of patients seen: demographics, education and income, skills and personality, common health and medical conditions.
3. **Threats to Health, Causes and Treatment**
   a. Treatment and recommendations to patients who need to make lifestyle changes to reduce their risk of chronic disease or severity if already ill.
   b. Main factors related to patients poor health lifestyles, motivations, and types of support needed for patient's to modify their behaviors.
4. **Health Information and Communication**
   a. What role does health information and communication in patients' behavior modification and self-care?
   b. Prior experiences of patients who used online information resources and tools.
      i. Examples of patients making use of the Internet, patient portals, EMRs and PHRs?
      ii. How did this help patients in making and/or maintaining behavior change?
      iii. How did this impact patients self-efficacy and confidence?
      iv. How did this impact the relationship between patient and provider?
   c. How has the provider used online health information resources or other forms of technology to promote behavior modification?
      i. Recommended information sources or specific online sites?
      ii. Recommended use of sites to track health status, body weight, physical activity, diet and nutrition?
      iii. Recommended patients review their electronic medical records?
      iv. Recommended self-tracking tools or health/medical apps for smartphones?
   d. Use of email, Twitter, or social media to communication with patients or to extend expertise to the wider community?
   e. Usefulness of social media for patients who share a health problem or medical condition?
5. **Clinic-based, personalized, digital health tool**
   a. Describe how such a tool might work to health care provider.
   b. Interest in using this system?
   c. General thoughts regarding this proposed system.
      i. Effective in promoting behavior change? Improving patient self-care?
      ii. Effective in improving patient knowledge?
      iii. Improve patient-provider communication and shared decision making?
   d. What would be critical for such a system to be widely adopted in clinics?
      i. Workflow issues? Ease of use?
      ii. Reimbursement?
      iii. Effectiveness in achieving outcomes?
   e. What would be critical for this system to be adopted and used by patients?
   f. Any concerns over privacy and security?

    g. Any concerns over patient skills and knowledge to make use of such a system?

6. **General recommendations, suggestions, advice regarding this proposed clinic-based information and communication system?**
7. **Closing -- Thank participant for their time and comments.**
